# Clinical Phenotypes of PCOS: a Cross-Sectional Study

**DOI:** 10.1007/s43032-023-01262-4

**Published:** 2023-05-22

**Authors:** Abdalla Moustafa Elsayed, Latifa Saad Al-Kaabi, Noora Mohammed Al-Abdulla, Moza Salem Al-Kuwari, Asmaa Abdulsamad Al-Mulla, Raghad Shaher Al-Shamari, Ahmed Khaled Alhusban, Ali Ahmed AlNajjar, Suhail A. R. Doi

**Affiliations:** 1 College of Medicine, QU Health, Qatar University, Doha, Qatar; 2 Department of Population Medicine, College of Medicine, QU Health, Qatar University, P.O. Box 2713, Doha, Qatar

**Keywords:** Polycystic ovary syndrome, Androstenedione, Hirsutism, Insulin resistance, Phenotype, Rotterdam criteria, Doi-Alshoumer classification

## Abstract

This cross-sectional study examines the Doi-Alshoumer PCOS clinical phenotype classification in relation to measured clinical and biochemical characteristics of women with polycystic ovary syndrome (PCOS). Two cohorts of women (Kuwait and Rotterdam) diagnosed with PCOS (FAI > 4.5%) were examined. These phenotypes were created using neuroendocrine dysfunction (IRMA LH/FSH ratio > 1 or LH > 6 IU/L) and menstrual cycle status (oligo/amenorrhea) to create three phenotypes: (A) neuroendocrine dysfunction and oligo/amenorrhea, (B) without neuroendocrine dysfunction but with oligo/amenorrhea, and (C) without neuroendocrine dysfunction and with regular cycles. These phenotypes were compared in terms of hormonal, biochemical, and anthropometric measures. The three suggested phenotypes (A, B, and C) were shown to be sufficiently distinct in terms of hormonal, biochemical, and anthropometric measures. Patients who were classified as phenotype A had neuroendocrine dysfunction, excess LH (and LH/FSH ratio), irregular cycles, excess A4, infertility, excess T, highest FAI and E2, and excess 17αOHPG when compared to the other phenotypes. Patients classified as phenotype B had irregular cycles, no neuroendocrine dysfunction, obesity, acanthosis nigricans, and insulin resistance. Lastly, patients classified as phenotype C had regular cycles, acne, hirsutism, excess P4, and the highest P4 to E2 molar ratio. The differences across phenotypes suggested distinct phenotypic expression of this syndrome, and the biochemical and clinical correlates of each phenotype are likely to be useful in the management of women with PCOS. These phenotypic criteria are distinct from criteria used for diagnosis.

**Supplementary Information:**

The online version contains supplementary material available at 10.1007/s43032-023-01262-4.

## Introduction

Polycystic ovary syndrome (PCOS) is a common but poorly defined heterogeneous clinical entity that frequently presents during adolescence and is the most common cause of menstrual irregularity and hirsutism [1]. PCOS is also considered a leading cause of infertility mainly due to dysfunctional ovaries. It is associated with hyperandrogenism, insulin resistance, and neuroendocrine dysfunction. In addition, PCOS increases the risk of several medical conditions, such as type 2 diabetes mellitus, cardiovascular disease, and endometrial carcinoma [2].

PCOS is a highly prevalent disorder of reproductive endocrinology in women. A meta-analysis that included 24 population studies across the globe assessed the prevalence of PCOS based on several diagnostic criteria [1]. Using the NIH criteria that classify PCOS according to the presence of hyperandrogenism and oligo/anovulation, the prevalence was 6% worldwide. Using the Rotterdam criteria or the Androgen Excess and PCOS (AE-PCOS) Society criteria based on the presence of two out of three features (hyperandrogenism, ovulatory dysfunction, and polycystic ovarian morphology) [2, 3], the prevalence was 10% in the MENA region [4]. In Qatar PCOS prevalence has been reported to be between 12 and 18% as per the NIH criteria.

The main pathogenesis in PCOS is not clear yet, but probably results from multiple inherited and non-inherited causes. Several main hypotheses include genetic factors [5] leading to ovarian or neuroendocrine dysfunction [6], abnormalities of intermediary metabolism [7] or complex interrelated mechanisms [8]. Today, the most accepted hypothesis is that neuroendocrine dysfunction leads to excess ovarian theca cell stimulation, with hyperinsulinemia sensitizing an abnormal ovary to excess LH [9]. The alternate hypothesis is that it is a primary ovarian disorder and that high LH (neuroendocrine dysfunction) is a consequence of this disorder [10]. With both hypotheses, there is ovarian dysfunction and there is some evidence that perhaps theca cells from ovaries of women with PCOS have elevated levels of a DENND1A splice variant (DENND1A.V2) which increases androgen biosynthesis [11].

Though PCOS is a syndrome with wide variability [3], there are few studies that have explored distinct phenotypes of PCOS. In 2012, the NIH came up with four PCOS phenotypes that are based on either two or all three of the Rotterdam criteria being present [12], but this has not been clinically useful, because several etiological factors may lead to any of these three criteria. The development of a clinically applicable phenotypic classification has proven difficult, even though the phenotypic approach has practical applications that can be utilized in routine clinical practice for treatment and prognosis. For instance, the phenotypic classification of PCOS patients based on the presence or absence of certain features may enable physicians to assess the patients’ risk for metabolic or reproductive dysfunction, which also determines the most suitable course of treatment. In addition, clinically relevant phenotypes may have importance for the genetics of PCOS, now that the instruments to identify the genes driving this polygenic disease are available [5].

In this study, we examine two large comprehensive databases of women with PCOS and examine possible phenotypes based on cycle status and neuroendocrine dysfunction, first suggested in 2008, by Doi and AlShoumer [13]. Three phenotypic groups were suggested, with phenotypes A and B being those with irregular cycles and phenotype C with regular cycles [13]. Of those with irregular cycles, phenotype A was defined as those with neuroendocrine dysfunction and phenotype B without neuroendocrine dysfunction [13]. If indeed there are three clinical phenotypes of the syndrome that represent different expressions of the same metabolic disorder, then these should be reflected by varying degrees of metabolic dysfunction, and, therefore, this study will examine the characteristics of a heterogeneous group of patients in an attempt to assess and validate the clinical utility of the Doi-Alshoumer clinical classification (as opposed to diagnostic classification) of PCOS.

## Methods

### Design and Data Sources

A cross-sectional design was used to assess the possible categorization of phenotypes for PCOS through the assessment of hormonal, biochemical, and anthropometric measures. These measures were looked at in terms of common patterns and then interpreted in terms of the existing best practice in management for each documented phenotype based on their observed characteristics.

### Patient Population

Data of women with PCOS were recruited from the previous practice of the senior author (SD) (referred to as Kuwait cohort in the study) [6, 7, 14], and this formed the primary dataset and had a total of 210 women that met our study criteria. Data were also analyzed from a dataset made publicly available by Krul-Poel et al. (referred to as Rotterdam cohort in the study) [15] and used to validate the findings from the first dataset and had a total of 310 women that met our study criteria.

#### Kuwait Cohort

To qualify as a PCOS index case, a woman had to have biochemical hyperandrogenism (FAI above 4.5%). This definition includes those women with regular menstrual cycles, even though hirsutism with regular menstrual cycles is frequently labeled as idiopathic hirsutism.

Secondary causes of hirsutism and anovulation, such as nonclassical 21-hydroxylase deficiency, hyperprolactinemia, or androgen-secreting tumors, were excluded by appropriate tests. Confounding medications that affect the metabolic criteria examined were excluded, and these include oral contraceptive agents, hypertensive medications, and insulin-sensitizing medications. Other confounding reproductive conditions, including pre-menarche, pregnancy, lactation, hysterectomy, or menopause, were also excluded.

#### Rotterdam Cohort

To qualify as a PCOS index case, women were screened for anovulatory infertility at the outpatient clinic of the Erasmus Center Rotterdam, and subsequently diagnosed with PCOS according to the Rotterdam criteria, i.e., requiring the presence of at least two out of the three following criteria: ovulatory dysfunction resulting in oligomenorrhea and/or amenorrhea, hyperandrogenism and/or hirsutism, and the presence of polycystic ovarian morphology (PCOM) [2].

Clinical and biochemical hyperandrogenism was defined as an FG score > 8, and/or a free androgen index (FAI > 4.5). Women were excluded if the blood draw was not performed in a fasting state.

### Hormonal and Computed Variables

Details of hormonal assays are given in the supplementary material. Body mass index (BMI) was calculated as the ratio of weight (kg)/height (m)^2^. Obesity was defined by the conventional cut-off of 30 kg/m^2^, since with BMI < 30 kg/m^2^, there is a greater incidence of inappropriate gonadotropin secretion [16] and less hyperinsulinemia (since insulin sensitivity decreases significantly in humans (without PCOS) above 27kg/m^2^) [17].

The homeostasis model assessment (HOMA) was applied for insulin sensitivity analysis and can be calculated from a computer program [18], available free from the Diabetes Trials Unit of the University of Oxford. These predictions allow the deduction of beta cell function (HOMA%B) and insulin sensitivity (HOMA%S) from pairs of fasting glucose and insulin (or C-peptide) measurements. Unlike fasting insulin (FI) and the insulin-glucose ratio (IGR), the HOMA calculation compensates for fasting hyperglycemia. The HOMA value correlates well with clamp techniques and has been frequently used to assess changes in insulin sensitivity after treatment [19]. HOMA was already computed for the women in the Rotterdam dataset.

The LH/FSH ratio was also used with a cut-off value of 1 because of the higher selectivity of our IRMA assay, as it has been documented that this brings the cut-off value between normal and PCOS patients down to equal or above 1. However, the conventional cut-off value for the LH/FSH ratio in the past has been 2 [20, 21]. Neuroendocrine dysfunction was defined as LH/FSH > 1 *or* LH > 6 IU/L. The P4 to E2 ratio was defined as follicular phase P4 (nmol/L) divided by follicular phase E2 converted to nmol/L (from pmol/L) [7].

### Clinical Assessment

#### Kuwait Cohort

All participants were examined between cycle days 1 and 7 after spontaneous menstruation or after prolonged amenorrhea. A regular menstrual cycle was defined as a cycle with an intermenstrual interval of 21–35 days, and the cycle length variation from one period to another was ≤ 7 days. A cycle was considered irregular if oligomenorrheic (intermenstrual interval ≥ 36 days (< 9 cycles per year)) or amenorrhoeic (intermenstrual interval > 6 months). Hirsutism was graded using a modified Ferriman and Gallwey scoring system [22] based on a study by Derksen et al. [23]. A woman was considered hirsute if the modified score was ≥ 4 counted from only five (instead of nine) hormone-sensitive areas (i.e., lip, chin, chest, upper abdomen, and lower abdomen) or ≥ 8 if the full score was used. Written informed consent was obtained from all subjects by the local ethics committee at the institutions where the data were collected.

#### Rotterdam Cohort

The authors reported that a thorough general medical, reproductive, and family history was taken, including self-reported ethnicity. Anthropometric measurements were performed, including height, weight, body mass index (BMI), systolic and diastolic blood pressure, and the level of hirsutism measured with the use of the Ferriman-Gallwey (FG) score. Finally, an extensive metabolic and endocrine profile was reported.

### Statistical Analysis

Descriptive statistics were used to describe the characteristics of the participants and included frequencies, percentages, and graphs. Continuous variables were summarized as the median and interquartile range (IQR). Three phenotypes were compared, and hormone levels were analyzed using ANOVA or the Kruskal-Wallis (non-parametric ANOVA) as appropriate for steroid hormones, fasting insulin, and gonadotropin levels across groups. The *χ*2-statistic was used to determine differences between groups for categorical variables. Reported *P* values should be interpreted as evidence against the model hypothesis at our sample size and interpreted in conjunction with the reported medians or means. We considered a *P* value below 0.001 as very strong evidence, between 0.001 and 0.01 as strong evidence, between 0.01 and 0.05 as moderate evidence, between 0.05 and 0.1 as weak evidence, and between 0.1 and 1 as little or no evidence against the model hypothesis at our sample size. All analyses were performed using Stata version 17.

## Results

### Kuwait Cohort

The Kuwait cohort had a total of 210 women across three phenotypes (A = 84, B = 54, C = 72). The mean ages of women in groups A, B, and C were similar and between 23 and 24 years, respectively. Most of the population were Kuwaiti Arabs in all three groups. Infertility was lowest in group C and highest in groups A and B (*P* = 0.001). Irregular menses were used to define the groups and thus present in groups A and B but not in group C. Acne (*P* = 0.16) and hirsutism (Ferriman Gallwey score; *P* = 0.009) were highest in group C even though FAI was highest in group A. Acanthosis was highest in group B and lowest in group C (*P* = 0.002). This also corresponds to the findings of HOMA2 IR (since acanthosis is exacerbated by insulin resistance). There was no difference in blood pressure between all groups.

**Fig. 1:**
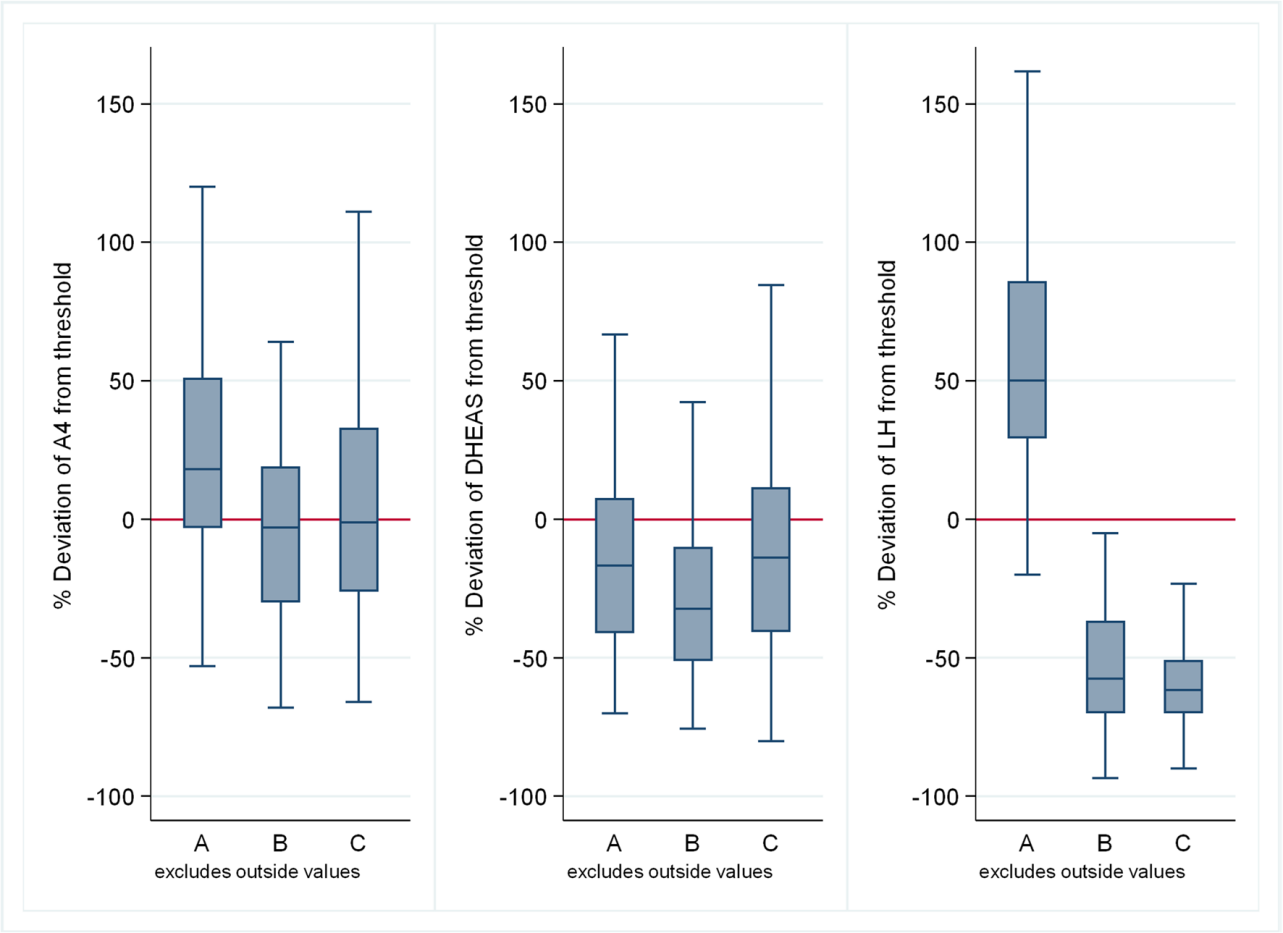
Distribution of hormonal values (from left to right panels: A4, DHEAS, LH) among PCOS phenotypes in terms of deviation from the threshold (the threshold is the value that distinguishes normal from elevated values) in the Kuwait cohort

Figure 1 depicts the key hormonal differences across phenotypes. The median values of the steroids A4 (*P* < 0.001), T (*P* = 0.017), 17αOHPG (*P* < 0.001), and E2 (*P* < 0.001) were all highest in group A with the median A4 and T in group A being higher than the normal value range (1.5–10.2 nmol/L and > 2.4 nmol/L, respectively). The only steroids that were highest in group C were median DHEAS, an adrenal steroid, and median P4. There were no steroids highest in group B. A4 (*P* < 0.001), 17αOHPG (*P* < 0.001), and DHEAS (*P* = 0.014) were all lowest in group B. Median T (*P* = 0.017), FAI (*P* = 0.001), and E2 (*P* < 0.001) were lowest in group C.

The median P4 to E2 ratio was significantly higher in group C and lowest in groups A and B (*P* < 0.001). This was despite a higher P4 level in the other groups suggesting that this was largely driven by E2 levels.

As expected, the median LH was significantly higher in group A and lower in groups B and C (*P* < 0.001). The median LH/FSH ratio was significantly higher in group A and lower in groups B and C (*P* < 0.001). This was expected as group A was defined by neuroendocrine dysfunction. The median SHBG was similar in all three groups (*P* = 0.14).

The median BMI was highest in group B and lowest in group C (*P* = 0.095), even though both group A and C medians were in the overweight category while group B was mostly obese class 1. Fasting blood glucose was almost similar between all groups (*P* = 0.35). Fasting insulin was significantly higher in group B and lowest in group C (*P* = 0.014). In keeping with the latter, median HOMA2 IR was highest in group B and lowest in group C (*P* = 0.015). The median HOMA2 %B was highest in group B and similar in groups A and C (*P* = 0.084). There was no difference in the median triglycerides (*P* = 0.18) and cholesterol (*P* = 0.89) levels between the groups.

### Rotterdam Cohort

The Rotterdam cohort had a total of 310 women across three phenotypes (A = 252, B = 39, C = 19). The mean ages of women in groups A, B, and C were similar and between 32 and 35 years. Most of the population were Caucasians in all three groups. Cycle intervals were used to define delayed cycles and thus present in groups A and B but not in group C. Ferriman Gallwey score (*P* = 0.41) was highest in group C even though FAI was highest in group A. There was no difference in blood pressure between all groups.

**Fig. 2:**
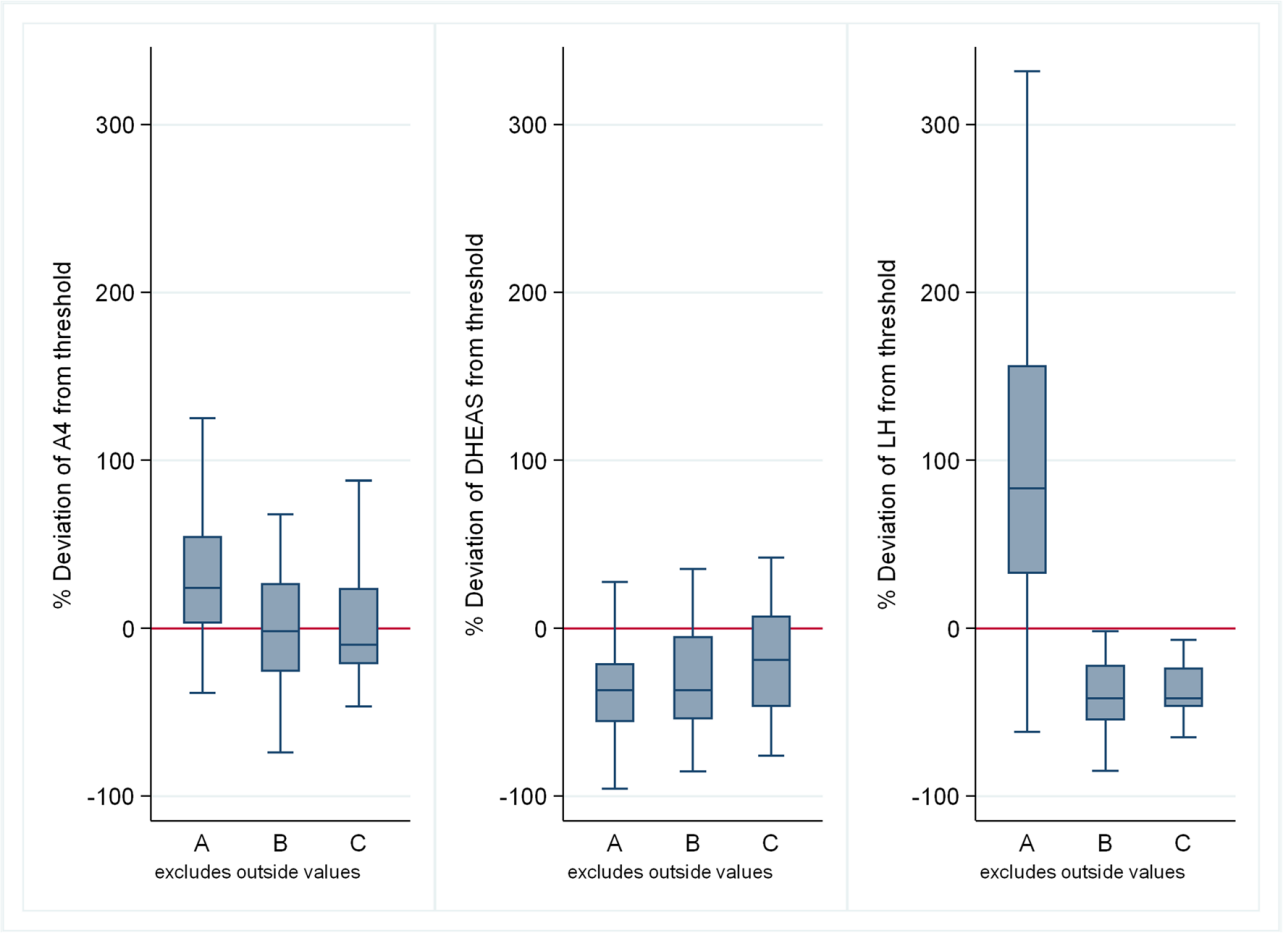
Distribution of hormonal values (from left to right panels: A4, DHEAS, LH) among PCOS phenotypes in terms of deviation from the threshold (the threshold is the value that distinguishes normal from elevated values) in the Rotterdam cohort

In this dataset, it can be noticed that most of the population is categorized as phenotype A. Fig. 2 depicts the key hormonal differences across phenotypes. The median values of the steroids A4 (*P* < 0.001), T (*P* < 0.001), 17αOHPG (*P* < 0.001), and E2 (*P* < 0.001) were all highest in group A with the median A4 and T in group A being higher than the normal value range (1.5–10.2 nmol/L and > 2.4nmol/L, respectively). The only steroid that was highest in group C was median DHEAS, an adrenal steroid. P4 levels were similar in all groups. There are no steroids that are highest in group B when compared to groups A and C.

T (*P* < 0.001), 17αOHPG (*P* < 0.001), and E2 (*P* < 0.001) were all lowest in group B, but median A4 (*P* < 0.001) was lowest in group C. However, DHEAS levels were equivalent in groups A and B and lower than group C. The median P4 to E2 ratio was significantly higher in group C and lowest in groups A and B (*P* = 0.002).

As expected, the median LH was significantly higher in group A and lower in groups B and C (*P* < 0.001), while the median LH/FSH ratio was significantly higher in group A and lower in groups B and C (*P* < 0.001) and this was expected as group A was defined by neuroendocrine dysfunction. The median SHBG (*P* = 0.039) was highest in group A and lowest in group B.

The median BMI was highest in group B and lowest in groups A and C (*P* = 0.026). Fasting blood glucose was almost similar between all groups (*P* = 0.29). Fasting insulin was higher in group B and lowest in group C (*P* = 0.2); this is supported by little or no evidence against the model hypothesis at this sample size. In keeping with the latter, the median HOMA2 IR was highest in group B and lower in groups A and C (*P* = 0.17). There was no difference in the median triglycerides (*P* = 0.67) and cholesterol (*P* = 0.75) levels between the groups.

### Summary of Phenotypic Findings

**Fig. 3:**
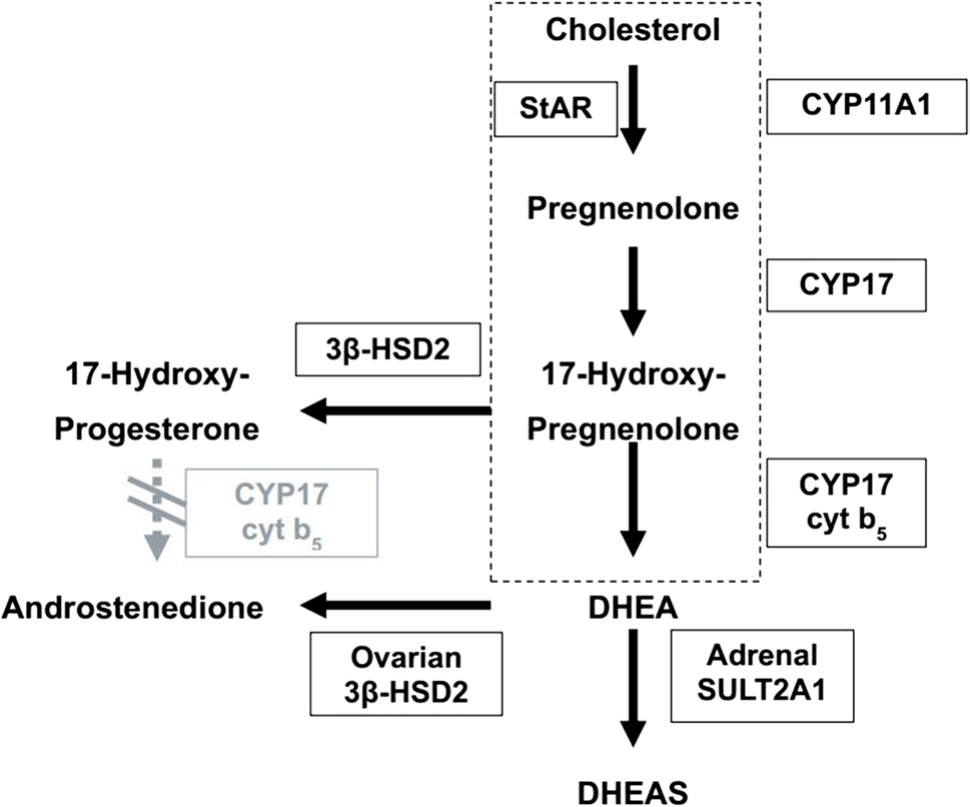
The absence of 3β-HSD in the adrenal reticularis means DHEA moves to DHEAS while its presence in the theca cell means that DHEA moves to A4. In the theca cell of normal women, there is no expression of 17β-HSD type 3 and hence, T is not produced. The pathway outlined by the rectangle is common in both the adrenal and the ovaries [adapted from Doi et al. [14]]

Table 1 summarizes the key differences between the phenotypes in terms of key features and includes suggested core pathways, biochemical pathways, putative genetics, and implications for practice. The key biochemical pathways involved are depicted in Fig. 3.

**Table 1:** Overview of the three clinical phenotypes in PCOS

	Subtypes		
	A	B	C
Key features	Neuroendocrine dysfunction; irregular cycles; excess LH (and LH/FSH ratio); excess A4; Infertility; excess T Highest FAI; highest E2 (it is ovarian); excess 17αOHPG	Irregular cycles and no neuroendocrine dysfunction; obesity; acanthosis; insulin resistance	Regular cycles; acne; hirsutism; excess P4; highest P4 to E2 molar ratio
Core pathways	Ovarian	Metabolic	Adrenal
Biochemical pathway	Increased 3β-HSD activity and increased ovarian E2	Decreased CYP17 and moderate decrease in ovarian and adrenal steroids due to insulin resistance with excess aromatase activity in adipose tissue and excess E2 Insulin enhances peripheral steroidogenesis, while inducing a relative impairment of CYP17 activity leading mainly to T excess without A4 and DHEAS excess[14]	Relatively insulin sensitive Decreased 3β-HSD activity or increased SULT2A1 or both. Most will have increased P4 to E2 molar ratio
Genetic pathway	DENND1A	Grb14 is negatively associated with insulin sensitivity	Not clear
Implications for practice	Ovarian steroid excess with less adrenal steroids Responds to ovarian ablation therapy that restores ovulation and fertility (14)	Not responsive to ovarian ablation therapy in terms of anovulatory cycles Better response to E2 decreasing or modulating therapies like letrozole and clomiphene for fertility	Ovarian ablation therapy not required as they are ovulatory More hirsute suggesting that adrenal androgens have a role to play in hirsutism Excess adrenal steroids with less ovarian steroids lead to excess hirsutism

## Discussion

Clinical, morphological, biochemical, endocrine, and, more recently, molecular studies have identified an array of underlying abnormalities which have added to the confusion concerning the pathophysiology of PCOS [8]. Despite the vast literature regarding the etiology and classification of PCOS, no consensus has been reached regarding the significance of features that manifest within the syndrome. For instance, the significance of elevated serum LH concentrations, insulin resistance, or polycystic-appearing ovaries assessed by ultrasound for PCOS remains uncertain. In contrast, hyperandrogenism and chronic anovulation generally are believed to be mandatory diagnostic features, at least until the Rotterdam consensus in 2003 [2].

In 2008, Doi and Alshoumer [13] first suggested three phenotypes of PCOS based on biochemical and clinical observations. They noted that serum FSH and E2 levels are usually found to be within the (broad) normal ranges, whereas LH may either be normal or elevated. Because PCOS with normal or high LH does seem to represent different clinical entities, they suggested that it seemed justifiable to consider these two subgroups distinct. They also observed that normal gonadotropins and normal A4 and E2 concentrations usually indicated the ovulatory form of the condition [7], whereas elevated gonadotropins indicated the anovulatory form of the condition [6]. Finally, they reported that many anovulatory patients who are obese may present with gonadotrophin levels within the normal range but retain elevated E2, suggesting yet another group within the spectrum of PCOS [14, 16]. These features then defined the features of the three phenotypes, which we confirm are distinct from the analysis of two cohorts of women with PCOS.

In this study, the phenotypic observations from the Kuwait cohort were replicated when applied to the Rotterdam cohort and this validates (for the first time) the phenotypic classification described. In addition to these two cohorts having similar profiles, there have been at least two other cohorts [24, 25], where this pattern of phenotypes was independently reproduced using cluster analysis. The first cluster study [25] delineated four clusters, with the first three resembling our phenotypes A, B, and C. They did not use cycle status for clustering but demonstrated that cluster 1 had high LH and a high proportion of irregular cycles, and cluster 2 did not have high LH but also had a high proportion of irregular cycles. The latter two clusters are consistent with phenotypes A and B. The third cluster had high DHEAS levels and a lower proportion of irregular cycles and was consistent with phenotype C. They also had a fourth cluster that were markedly insulin-resistant and probably represent an obese subset of phenotype B. In the second study [24] that used cluster analysis, three clusters were reported: a reproductive cluster with high LH that was consistent with phenotype A, a metabolic cluster with insulin resistance that was consistent with phenotype B, and an indeterminate cluster that had some similarities with phenotype C.

The mechanism of neuroendocrine dysfunction in phenotype A may be an uncoupling of hypothalamic E2 inhibition by elevated ovarian A4 [6]. This abnormal secretion of ovarian A4 is an intrinsic property of PCOS theca cells [26] and, contrary to the conventional view, this then leads to excess LH, with the expected excess E2-related supression of pituitary LH release being opposed by excess A4 and this is what then drives the increase in LH secretion in this phenotype. There is also excess ovarian E2 that, again contrary to the conventional view, is likely responsible for menstrual irregularity and possibly anovulation in PCOS[7]. The putative mechanism is likely the excess E2 leading to a loss of the P4 modulation of the E2 effect on the ovaries leading to inhibition of FSH receptor signal transduction [7]. This is also supported by the demonstration that the P4/E2 molar ratio correlates quite well with ovulation in PCOS but *not* gonadotropin or androgen levels [7]. In addition, a report of two women with features of phenotype A shows that ovarian ablative therapy restores ovulation and was associated with lower E2 levels [27] lending further support to the excess ovarian E2 hypothesis. This phenotype could be linked to the ovarian steroid synthesis pathway through a genetic mutation in DENND1A that may drive the overexpression of 3β-HSD, which affects the increased theca cell production of androgens [24, 28] and subsequently E2.

The increase in insulin resistance (and obesity) in phenotype B is associated with decreased A4 synthesis, both of which decrease LH secretion independently of each other. Here, the excess E2 is predominantly extraovarian, presumably from adipose tissue conversion of androgens, and thus, a hallmark of phenotype B is evidence of obesity, cycle irregularity, and the expectation that this would not respond to ovarian ablative therapy [29], given that the excess E2 is mainly extraovarian, and therefore, this procedure will not lower E2 levels. It should be noted that many studies are inconsistent about changes in E2 after ovarian ablative therapy [30], and this likely reflects a mix of phenotypes A and B being examined in these studies. This observation seems to be the basis of many reports suggesting that anovulatory women who are lean (mainly ovarian E2) do well with ovarian E2 reducing therapies such as ovarian cauterization [31–34], while obese anovulatory women (with peripheral E2 production) usually fare better with FSH increasing therapies via gonadotropins or clomiphene [35–37]. In this phenotype, the main approach to management then seems to be to increase insulin sensitivity either via insulin sensitizers, for example, metformin, or weight loss. This will decrease the increased extraovarian E2 synthesis and, in conjunction with clomiphene citrate, which acts as an estrogen receptor modulator in the hypothalamus and pituitary, will minimize the negative inhibition of E2 leading to the facilitation of ovulation and fertility. Also, patients can be treated with letrozole, which is an aromatase inhibitor preventing the conversion of T to E2, again facilitating ovulation. In this group, a mutation in the region of the Grb14 gene has been suggested to be what drives insulin resistance [24, 38], given that Grb14 is an effector of insulin signaling and directly inhibits insulin receptor catalytic activity in vitro. It has also been shown that prolonged fasting and treatment with metformin significantly decreased Grb14 expression in peri-epididymal adipose tissue [39].

In phenotype C, excess adrenal steroidogenesis, as evidenced by the highest DHEAS levels, was associated with the highest molar ratios of P4 to E2. Although P4 synthesis is adrenal in the follicular phase of women [40, 41], the main driver here seems to be the lowest E2 levels in the follicular phase in those with phenotype C. It is unclear what drives adrenal hyperandrogenism in this group. In a study by Carbunaru et al. [42], hyperandrogenic females (HF) with decreased adrenal 3β-HSD activity were compared with classic PCOS. They found that insulin sensitivity and gonadotropin data in both HF with the descending 3β-HSD phenotype and classic PCOS indicated significant insulin resistance and LH hypersecretion in both suggesting that the descending 3β-HSD phenotype in HF is probably a variant of PCOS. The LH/FSH ratio was 1.1 ± 0.24 in HF with decreased adrenal 3β-HSD activity. There was not much data on the specific cycle status of these patients. It is possible that this group of HF with decreased 3β-HSD activity and excess DHEAS corresponds to phenotype C in our study while the group of HF with classic PCOS may correspond to a combination of phenotype clusters A and B [42]. Carbunaru et al. found no evidence of an adrenal mutation that can drive congenital adrenal hyperplasia. It is well-known that having DHEAS excess in HF could also mean that there could be an adrenal mutation of 3β-HSD gene (nonclassical congenital adrenal hyperplasia). However, Carbunaru et al. [42] found no evidence of such mutations and concluded that the perceptible decrease in 3β-HSD was ovarian, not adrenal. We interpret this as the excess ovarian DHEA not converted to A4 and being increasingly converted to DHEAS in the adrenal gland and the excess DHEAS being a marker of decreased production of A4 as well as E2. These patients are therefore likely to remain ovulatory and the main management need for this group is symptomatic treatment such as spironolactone for hirsutism [42].

These clinical phenotypes differ from the diagnostic classification suggested by the NIH and Rotterdam criteria (Table 2), which do not give adequate insight into any of the special characteristics of each phenotype. Both these criteria only provided data on the diagnosis of PCOS without focus on special characteristics of the disease and how they differ from one patient to the other. The diagnostic criteria are therefore distinct from the phenotypic classification suggested in this paper (Table 2), and this is understandable since only one of the NIH or Rotterdam criteria (oligo/amenorrhea) [43] is included in the definition of these clinical phenotypes. In the Rotterdam cohort, phenotype A predominated because selection into this cohort was based on these diagnostic criteria that excluded some of the women in phenotypes B and C. We reclassified some of the excluded women for this study based on our own inclusion criteria for the Kuwait cohort. Although elevated LH is not part of the Rotterdam criteria, some countries like Japan have included this in their diagnostic criteria [44] where PCOS is defined when three criteria are met: oligo/amenorrhea, polycystic ovaries, and high levels of serum androgens or LH.

**Table 2:** PCOS classification

Classification into clinical phenotypes (Doi-Alshoumer)	
Group	Criteria
A	Neuroendocrine dysfunction (IRMA LH/FSH ratio >1 or LH >6 IU/L) and irregular menstrual cycles (oligo/amenorrhea)
B	Without neuroendocrine dysfunction but with irregular menstrual cycles (oligo/amenorrhea)
C	Without neuroendocrine dysfunction and with regular menstrual cycles
Diagnostic criteria (Rotterdam)	
Presence of two of three of the following criteria: 1—Oligo/amenorrhea 2—Hyperandrogenism 3—Polycystic ovaries (≥ 12 follicles measuring 2–9 mm in diameter and/or an ovarian volume > 10 mL in at least one ovary)	

 It is now obvious that the majority of women labeled to have idiopathic hirsutism also have polycystic ovaries by ultrasound and at least one endocrine abnormality to support the diagnosis of PCOS [45]. Further studies also reveal the same abnormalities of ovarian steroidogenesis in these women as in classic PCOS, suggesting that mild PCOS is much more common than idiopathic hirsutism [46]. Indeed, about half of the hyperandrogenic sisters of probands with chronic hyperandrogenic anovulation themselves have ovulatory menstrual cycles [26], and so-called idiopathic hirsutism with normo-androgenemia still has subtle increases in the ovarian secretion of 17αOHPG and a minimally increased adrenal (Δ4) 17, 20-lyase activity. This suggests that even these patients themselves might represent the mildest forms of PCOS [47]. Women who have both regular menstrual cycles and normal serum androgens (FAI) but the presence of clinical hyperandrogenism (hirsutism) might represent the mildest form of PCOS but were excluded from this series as their metabolic profile may be similar to normal women and distinct from the metabolic profile of women in this series, including phenotype C (Table 3).

**Table 3:** Summary of metabolic profiles in the three clinical phenotypes

Factor	A	B	C	*p* value*
Kuwait				
*N*	84	54	72	
A4 (nmol/L)	11.8 (9.7, 15.1)	9.7 (7.0, 11.9)	9.9 (7.4, 13.3)	< 0.001
DHEAS (μmol/L)	7.5 (5.3, 9.7)	6.1 (4.4, 8.1)	7.8 (5.4, 10.0)	0.014
T (nmol/L)	2.8 (2.2, 3.7)	2.4 (1.8, 3.3)	2.5 (1.8, 3.2)	0.017
FAI (%)	15.1 (10.8, 21.6)	12.3 (8.3, 20.7)	11.7 (6.6, 16.5)	0.001
P4 (nmol/L)	4.6 (3.8, 5.7)	4.4 (3.7, 5.3)	5.2 (4.1, 7.3)	0.17
17αOHPG (nmol/L)	7.8 (5.3, 10.1)	4.8 (3.4, 8.0)	6.1 (4.0, 8.9)	< 0.001
E2 (pmol/L)	154.5 (118.5, 194.5)	114.0 (73.0, 167.0)	110.0 (83.0, 152.0)	< 0.001
P4 to E2 molar ratio	28.4 (22.7, 35.3)	27.8 (21.6, 37.1)	48.0 (38.3, 71.4)	< 0.001
BMI	29 (26, 36)	32 (27, 37)	28 (24, 35)	0.095
HOMA2 IR	1.7 (1.0, 3.3)	2.3 (1.8, 3.2)	1.3 (1.0, 2.0)	0.015
Rotterdam				
*N*	252	39	19	
A4 (nmol/L)	12.4 (10.3, 15.5)	9.8 (7.4, 12.7)	9.0 (7.9, 12.4)	< 0.001
DHEAS (μmol/L)	5.7 (4.0, 7.1)	5.7 (4.1, 8.6)	7.3 (4.8, 9.7)	0.064
T (nmol/L)	2.5 (2.0, 3.2)	1.6 (1.4, 2.4)	1.8 (1.5, 2.1)	< 0.001
FAI (%)	8.1 (5.8, 11.5)	6.9 (5.3, 8.9)	6.9 (5.1, 8.9)	0.032
P4 (nmol/L)	1.3 (0.9, 2.1)	1.4 (1.0, 2.0)	1.4 (0.8, 1.9)	0.9
17αOHPG (nmol/L)	3.2 (2.4, 4.4)	1.8 (1.5, 2.2)	2.2 (1.5, 2.8)	< 0.001
E2 (pmol/L)	253.5 (203.5, 352.5)	165.0 (137.0, 208.0)	199.0 (122.0, 247.0)	< 0.001
P4/E2 molar ratio	5.5 (3.7, 8.3)	7.6 (6.1, 10.0)	8.0 (3.7, 17.0)	0.002
BMI	28 (24, 32)	31 (27, 35)	26 (24, 33)	0.026
HOMA2 IR	1.9 (1.1, 3.0)	2.5 (1.4, 3.5)	1.8 (1.2, 2.9)	0.17

Median (IQR) reported; *from Kruskal-Wallis test

## Conclusion

To date, a clear distinction between PCOS phenotypes has not been well established in the literature. With PCOS being the most researched reproductive endocrinology topic, the phenotypic classification represents a gap in the literature leading to unnecessary further tests or treatment measures and a lower quality of life for patients with PCOS [3]. The suggestion of three phenotypes in this study based on their underlying pathophysiology is supported by two previous cluster analyses and our biochemical analysis here. Our distinct phenotypes may assist researchers to define participants better in future PCOS research and may yield more specific outcomes. These phenotypes will also benefit clinical practice and patient outcomes. Further study and correlation with patient-important outcomes can ultimately help guide better-targeted treatment for patients with PCOS. We need further research to better understand how treatments interact with the phenotypes and what the best choices are for each phenotype. Further studies need to consolidate the genetic and biochemical differences among the phenotypes and correlate them with the different treatment modalities, thus allowing for better-targeted treatments for women with PCOS.

### Supplementary Information

ESM 1

## Data Availability

All data generated or analyzed during this study are available from the corresponding author on reasonable request.

## Abbreviations

17αOHPG: 17α-Hydroxyprogesterone
A4: Androstenedione
DHEAS: Dehydroepiandrosterone sulfate
E2: Estradiol
FSH: Follicle-stimulating hormone
GnRH: Gonadotrophin-releasing hormone
LH: Luteinizing hormone
P4: Progesterone
PCOS: Polycystic ovary syndrome
T: Testosterone
FAI: Free androgen index
HF: Hyperandrogenic female
FPG: Fasting plasma glucose
3β-HSD: 3-Beta-hydroxysteroid dehydrogenase
17β-HSD: 17-Beta-hydroxysteroid dehydrogenase
