# Supplementary material for: Clinical Phenotypes of PCOS: a Cross-Sectional Study
Source: Reprod Sci. 2023 May 22;30(11):3261–72. doi: 10.1007/s43032-023-01262-4 (PMC10643327; doi:10.1007/s43032-023-01262-4)
Supplement: Supplementary file 1 — ESM 1 [file 43032_2023_1262_MOESM1_ESM.docx]

**Supplementary Material**

1. **Assays and reference ranges**

**Kuwait cohort**

Serum LH (<6.2 IU/l) and FSH (<8.8 IU/l) were measured by coated tube immunoradiometric assay (IRMA) from Diagnostic Products Corp. (CA, USA). Sex hormone-binding globulin (SHBG, >20 nmol/l) was measured by a non-competitive ‘liquid phase’ IRMA from Orion Diagnostica (Espoo, Finland). Prolactin (<406 mIU/l) was measured by coated tube IRMA (DiaSorin, s.r.l., Saluggia, Italy). A4 (<9.2 nmol/l) was measured by radioimmunoassay (Diagnostic Systems Laboratories Inc., Texas, USA). Serum FAI (<3 nmol/l) and E2 (E_2_, <285 pmol/l) were measured by coated tube radioimmunoassay (Orion Diagnostica). Serum dehydroepiandrosterone sulphate (DHEA-S, <10.3 μmol/l), 17HP (<7.9 nmol/l), P4 (<4.5 nmol/l) and fasting insulin (FI, <22 mIU/l) levels were measured by a coated tube radioimmunoassay from Diagnostic Products Corp. Serum insulin-like growth factor I (IGF-I) levels were measured using a two-site IRMA from Diagnostic Systems Laboratories, Inc. The inter- and intra-assay variations of these assays were <10 and <5% respectively. All assays were highly specific with <1.4% cross-reactivity to structurally related hormones. None of the hormone assays included an extraction step to separate it from its binding protein in serum.

**Rotterdam cohort**

serum assays of FSH, PRL, TSH, LH, E2, A4 (AD), FAI (T), sex hormone-binding globulin (SHBG), cortisol, and dehydroepiandrosterone sulfate. Serum LH and FSH levels were measured by immunoradiometric assay (Medgenix, Fleurus, Belgium), and T, AD, SHBG, and dehydroepiandrosterone sulfate were determined using RIA kits (Diagnostic Products Corp., Los Angeles, CA). Liquid-liquid extraction (hexane) of the samples and analysis, carried out with a Waters ACQUITY UPLC system couple to a Waters Xevo TQ mass spectrometer, were performed as described elsewhere (19) Intra-assay coefficients of variation (CV) were < 6% and intra-assay CV was < 9% at the concentration of 16 and 80 nmol/L, respectively. The measurement of serum 25(OH)D was carried out by the central chemical laboratory of the Medical Center Alkmaar, the Netherlands. This laboratory is a (ISO-15189) certified laboratory. Endocrine evaluation included serum levels of gonadotropic hormones (LH, FSH) and estradiol (E2), testosterone, dehydroepiandrosterone sulfate (DHEAS), fasting glucose and insulin. Hormone assays have been described in detail elsewhere (20). Insulin was measured by immunoradiometric assay. Testosterone, E2 and DHEAS were determined by radioimmunometric assays (RIAs). Intra-assay and inter-assay CV were less than 3% and less than 5% for testosterone and less than 5% and less than 7% for E2 respectively.

**Hormonal Assays**

Kuwait cohort

All hormone assays were determined from one sample after an 8-hour overnight fast. This sample was obtained between 08:00 and 11:00 in the first week of the cycle or after more than 1 month of amenorrhea. Serum steroid hormone levels were determined as previously described[14].

Rotterdam cohort

For hormone assays venous blood samples were drawn at examination and stored at −80˚C after centrifugation at 3000 rpm for 10 min at 20˚C. Details are given in the supplementary material.

Steroid hormone assays in both datasets were run using immunoradiometric assays. Details are given in the supplementary material. Assays were run immediately in the Kuwait dataset, while in the Rotterdam dataset, venous blood samples were centrifuged within 2 hours after withdrawal and were stored at -80˚C.

1. **PCOS Profile - Kuwait cohort**

**Table S1: steroid hormones**

| **Factor** | **A** | **B** | **C** | **p-value*** |
| --- | --- | --- | --- | --- |
| **N** | 84 | 54 | 72 |  |
| **A4 (nmol/L)** | 11.8 (9.7, 15.1) | 9.7 (7.0, 11.9) | 9.9 (7.4, 13.3) | <0.001 |
| **DHEAS (µmol/L)** | 7.5 (5.3, 9.7) | 6.1 (4.4, 8.1) | 7.8 (5.4, 10.0) | 0.014 |
| **T (nmol/L)** | 2.8 (2.2, 3.7) | 2.4 (1.8, 3.3) | 2.5 (1.8, 3.2) | 0.017 |
| **FAI (%)** | 15.1 (10.8, 21.6) | 12.3 (8.3, 20.7) | 11.7 (6.6, 16.5) | 0.001 |
| **P4 (nmol/L)** | 4.6 (3.8, 5.7) | 4.4 (3.7, 5.3) | 5.2 (4.1, 7.3) | 0.17 |
| **17αOHPG** **(nmol/L)** | 7.8 (5.3, 10.1) | 4.8 (3.4, 8.0) | 6.1 (4.0, 8.9) | <0.001 |
| **E2 (pmol/L)** | 154.5 (118.5, 194.5) | 114.0 (73.0, 167.0) | 110.0 (83.0, 152.0) | <0.001 |
| **P4 to E2 molar ratio** | 28.4 (22.7, 35.3) | 27.8 (21.6, 37.1) | 48.0 (38.3, 71.4) | <0.001 |
| **Cortisol (nmol/L)** | 434.0 (307.0, 541.0) | 408.0 (266.0, 565.0) | 358.0 (263.0, 529.0) | 0.46 |

Median (IQR) reported; *From Kruskal-Wallis test.

**Table S2: other hormones**

| **Factor** | **A** | **B** | **C** | **p-value*** |
| --- | --- | --- | --- | --- |
| **N** | 84 | 54 | 72 |  |
| **LH (IU/L)** | 9.0 (7.8, 11.1) | 2.5 (1.8, 3.8) | 2.3 (1.8, 3.0) | <0.001 |
| **FSH (IU/L)** | 5.9 (4.9, 6.9) | 5.2 (4.2, 5.7) | 5.3 (4.4, 6.6) | 0.003 |
| **LH to FSH ratio** | 1.5 (1.2, 2.0) | 0.6 (0.4, 0.7) | 0.4 (0.3, 0.6) | <0.001 |
| **SHBG (nmol/L)** | 19.0 (13.0, 26.0) | 19.0 (14.0, 24.0) | 22.0 (15.8, 31.0) | 0.14 |

Median (IQR) reported; *From Kruskal-Wallis test.

**Table S3: intermediary metabolism (glucose homeostasis)**

| **Factor** | **A** | **B** | **C** | **p-value*** |
| --- | --- | --- | --- | --- |
| **N** | 84 | 54 | 72 |  |
| **BMI** | 29 (26, 36) | 32 (27, 37) | 28 (24, 35) | 0.095 |
| **FPG** | 5.3 (5.0, 5.8) | 5.3 (5.1, 5.5) | 5.2 (4.9, 5.6) | 0.35 |
| **Fasting insulin (pmol/L)** | 86.4 (58.8, 168.0) | 123.0 (93.6, 188.4) | 73.2 (55.2, 102.0) | 0.014 |
| **HOMA2 IR** | 1.7 (1.0, 3.3) | 2.3 (1.8, 3.2) | 1.3 (1.0, 2.0) | 0.015 |
| **HOMA2 %B** | 105.4 (93.9, 175.1) | 149.4 (114.3, 203.4) | 110.8 (90.8, 141.2) | 0.084 |
| **Triglyceride (mmol/L)** | 1.0 (0.6, 1.7) | 0.9 (0.6, 1.2) | 0.8 (0.5, 1.4) | 0.18 |
| **Cholesterol (mmol/L)** | 4.6 (4.0, 5.1) | 4.6 (4.0, 5.2) | 4.5 (4.1, 5.1) | 0.89 |

Median (IQR) reported; *From Kruskal-Wallis test.

**Table S4: clinical and demographic data by phenotype**

| **Factor** | **Level** | **A** | **B** | **C** | **p-value*** |
| --- | --- | --- | --- | --- | --- |
| **N** |  | 84 | 54 | 72 |  |
| **Age^a^** |  | 23 (6) | 23 (6) | 22 (7) | 0.72 |
| **Ethnicity** | Other Arab | 21 (25.3%) | 7 (13.0%) | 10 (14.1%) | 0.033 |
|  | Asian | 10 (12.0%) | 9 (16.7%) | 3 (4.2%) |  |
|  | Kuwaiti Arab | 52 (62.7%) | 38 (70.4%) | 58 (81.7%) |  |
| **Fertility status** | Fertile | 6 (7.5%) | 9 (17.3%) | 12 (17.4%) | 0.001 |
|  | Infertile | 19 (23.8%) | 9 (17.3%) | 1 (1.4%) |  |
|  | Single | 55 (68.8%) | 34 (65.4%) | 56 (81.2%) |  |
| **Cycle interval** | ≥180 days | 7 (8.3%) | 5 (9.3%) | 0 (0.0%) | <0.001 |
|  | ≥36 days | 77 (91.7%) | 49 (90.7%) | 0 (0.0%) |  |
|  | <36 days | 0 (0.0%) | 0 (0.0%) | 72 (100.0%) |  |
| **Acne** |  | 18 (30.0%) | 11 (31.4%) | 22 (46.8%) | 0.16 |
| **Hirsutism** |  | 69 (83.1%) | 45 (83.3%) | 72 (100.0%) | 0.001 |
| **Ferriman-Gallwey score^b^** |  | 7 (4, 11) | 7 (4, 10) | 10 (6, 13) | 0.009 |
| **Acanthosis nigricans** |  | 14 (19.4%) | 19 (39.6%) | 7 (11.5%) | 0.002 |
| **BP(systolic)^a^** |  | 130 (17) | 129 (11) | 131 (30) | 0.95 |
| **BP(diastolic)^a^** |  | 85.2 (11.9) | 88.5 (12.0) | 85.4 (18.5) | 0.7 |

*From ANOVA, Pearson’s chi squared or Kruskal-Wallis test as appropriate

^a^mean (SD); ^b^median (IQR)

1. **PCOS profile - Rotterdam cohort**

**Table S5: steroid hormones**

| **Factor** | **A** | **B** | **C** | **p-value*** |
| --- | --- | --- | --- | --- |
| **N** | 252 | 39 | 19 |  |
| **A4 (nmol/L)** | 12.4 (10.3, 15.5) | 9.8 (7.4, 12.7) | 9.0 (7.9, 12.4) | <0.001 |
| **DHEAS (µmol/L)** | 5.7 (4.0, 7.1) | 5.7 (4.1, 8.6) | 7.3 (4.8, 9.7) | 0.064 |
| **T (nmol/L)** | 2.5 (2.0, 3.2) | 1.6 (1.4, 2.4) | 1.8 (1.5, 2.1) | <0.001 |
| **FAI (%)** | 8.1 (5.8, 11.5) | 6.9 (5.3, 8.9) | 6.9 (5.1, 8.9) | 0.032 |
| **P4 (nmol/L)** | 1.3 (0.9, 2.1) | 1.4 (1.0, 2.0) | 1.4 (0.8, 1.9) | 0.9 |
| **17αOHPG** **(nmol/L)** | 3.2 (2.4, 4.4) | 1.8 (1.5, 2.2) | 2.2 (1.5, 2.8) | <0.001 |
| **E2 (pmol/L)** | 253.5 (203.5, 352.5) | 165.0 (137.0, 208.0) | 199.0 (122.0, 247.0) | <0.001 |
| **P4/E2 molar ratio** | 5.5 (3.7, 8.3) | 7.6 (6.1, 10.0) | 8.0 (3.7, 17.0) | 0.002 |
| **Cortisol (nmol/L)** | 304.5 (227.5, 398.5) | 312.0 (255.0, 439.0) | 345.0 (224.0, 395.0) | 0.8 |

Median (IQR) reported; *From Kruskal-Wallis test.

**Table S6: Other hormones**

| **Factor** | **A** | **B** | **C** | **p-value*** |
| --- | --- | --- | --- | --- |
| **N** | 252 | 39 | 19 |  |
| **LH (IU/L)** | 11.0 (8.0, 15.4) | 3.5 (2.7, 4.7) | 3.5 (3.2, 4.6) | <0.001 |
| **FSH (IU/L)** | 6.2 (5.1, 7.3) | 6.2 (5.1, 7.1) | 5.8 (4.7, 7.0) | 0.49 |
| **LH to FSH ratio** | 1.8 (1.4, 2.4) | 0.6 (0.4, 0.7) | 0.6 (0.5, 0.8) | <0.001 |
| **SHBG (nmol/L)** | 28.8 (20.9, 39.6) | 22.2 (16.5, 33.6) | 24.5 (16.8, 35.2) | 0.039 |

Median (IQR) reported; *From Kruskal-Wallis test.

**Table S7: Intermediary metabolism (glucose homeostasis)**

| **Factor** | **A** | **B** | **C** | **p-value*** |
| --- | --- | --- | --- | --- |
| **N** | 252 | 39 | 19 |  |
| **BMI** | 28 (24, 32) | 31 (27, 35) | 26 (24, 33) | 0.026 |
| **FPG** | 4.4 (3.9, 4.8) | 4.6 (4.0, 5.1) | 4.2 (4.0, 5.1) | 0.29 |
| **Fasting insulin (pmol/L)** | 66.0 (40.5, 105.0) | 84.0 (52.0, 115.0) | 70.0 (43.0, 88.0) | 0.2 |
| **HOMA2 IR** | 1.9 (1.1, 3.0) | 2.5 (1.4, 3.5) | 1.8 (1.2, 2.9) | 0.17 |
| **Triglyceride (mmol/L)** | 1.0 (0.8, 1.4) | 1.0 (0.8, 1.7) | 0.9 (0.7, 1.4) | 0.67 |
| **Cholesterol (mmol/L)** | 5.0 (4.2, 5.9) | 4.8 (4.4, 5.5) | 5.0 (4.3, 6.2) | 0.75 |

Median (IQR) reported; *From Kruskal-Wallis test.

**Table S8: Demographic & clinical features**

| **Factor** | **Level** | **A** | **B** | **C** | **p-value*** |
| --- | --- | --- | --- | --- | --- |
| **N** |  | 252 | 39 | 19 |  |
| **Age^a^** |  | 33 (5) | 32 (5) | 35 (5) | 0.2 |
| **Ethnicity** | Caucasian | 130 (51.6%) | 18 (46.2%) | 9 (47.4%) | 0.4 |
|  | Mediterranean | 46 (18.3%) | 6 (15.4%) | 8 (42.1%) |  |
|  | Black | 32 (12.7%) | 5 (12.8%) | 1 (5.3%) |  |
|  | Asian (other than Indian) | 11 (4.4%) | 3 (7.7%) | 0 (0.0%) |  |
|  | Indian | 20 (7.9%) | 5 (12.8%) | 1 (5.3%) |  |
|  | Other | 13 (5.2%) | 2 (5.1%) | 0 (0.0%) |  |
| **Cycle interval** | ≥180 days | 88 (34.9%) | 16 (41.0%) | 0 (0.0%) | <0.001 |
|  | ≥36 days | 164 (65.1%) | 23 (59.0%) | 0 (0.0%) |  |
|  | <36 days | 0 (0.0%) | 0 (0.0%) | 19 (100.0%) |  |
| **Hirsutism** |  | 78 (32.4%) | 14 (35.9%) | 6 (31.6%) | 0.9 |
| **Ferriman-Gallwey score^b^** |  | 4 (1, 9) | 4 (1, 14) | 6 (4, 10) | 0.41 |
| **BP(systolic)^a^** |  | 119 (13) | 121 (12) | 119 (19) | 0.62 |
| **BP(diastolic)^a^** |  | 78.1 (10.9) | 81.7 (12.5) | 79.6 (12.1) | 0.16 |

*From ANOVA, Pearson’s chi squared or Kruskal-Wallis test as appropriate

^a^mean (SD); ^b^median (IQR)
